# Supplementary material for: Genomic and Transcriptomic Characterisation of Response to Neoadjuvant Chemoradiotherapy in Locally Advanced Rectal Cancer
Source: Cancers (Basel). 2020 Jul 6;12(7):1808. doi: 10.3390/cancers12071808 (PMC7408989; doi:10.3390/cancers12071808)
Supplement: Supplementary file 1 [file cancers-12-01808-s001.pdf]

cancers

MDPI

Article

Genomic and Transcriptomic Characterisation of Response to Neoadjuvant Chemoradiotherapy in Locally Advanced Rectal Cancer

Sinead Toomey [1,†], Jillian Gunther [2,†], Aoife Carr [1], David C. Weksberg [2,3], Valentina Thomas [4,5], Manuela Salvucci [5], Orna Bacon [5], El-Masry Sherif [6,7], Joanna Fay [8], Elaine W. Kay [8], Katherine M. Sheehan [8], Deborah A. McNamara [7], Keith L Sanders [2], Geena Mathew [2], Oscar S. Breathnach [9], Liam Grogan [9], Patrick G. Morris [9], Wai C. Foo [10], Yi-Qian N. You [11], Jochen H. Prehn [5], Brian O'Neill [12], Sunil Krishnan [2,*,‡], Bryan T. Hennessy [1,9,*] and Simon J. Furney [4,5,*]

[1] Medical Oncology Group, Department of Molecular Medicine, Royal College of Surgeons in Ireland, Dublin, Dublin 9, Ireland; sineadtoomey@rcsi.ie (S.T.); aoifecarr@rcsi.ie (A.C.); bryanhennessy74@gmail.com (B.T.H.)

[2] Department of Radiation Oncology, The University of Texas MD Anderson Cancer Center, Houston, TX 77030, USA; JGunther@mdanderson.org (J.G.); davidweks@gmail.com (D.C.W.); KLSanders@mdanderson.org (K.L.S); geenag@mdanderson.org (G.M.); krishnan.sunil@mayo.edu (S.K.)

[3] UPMC Pinnacle, Harrisburg, PA 17101, USA

[4] Genomic Oncology Research Group, Department of Physiology and Medical Physics, Royal College of Surgeons in Ireland, Dublin, Dublin 2, Ireland; valentinathomas@rcsi.ie (V.T.); simonfurney@rcsi.ie (S.J.F.)

[5] Centre for Systems Medicine, Department of Physiology and Medical Physics, Royal College of Surgeons in Ireland, Dublin, Dublin 2, Ireland; manuelasalvucci@rcsi.ie (M.S.); ornabacon@rcsi.ie (O.B.); JPrehn@rcsi.ie (J.H.P.)

[6] Department of Surgery, Our Lady of Lourdes Hospital Drogheda, Co. Louth, Ireland; selmasry@rcsi.ie

[7] Department of Surgery, Beaumont Hospital, Dublin, Dublin 9, Ireland; deborahmcnamara@rcsi.ie

[8] Department of Pathology, Royal College of Surgeons in Ireland, Dublin, Dublin 9, Ireland; joannafay@rcsi.ie (J.F.); elainewkay@gmail.com (E.W.K.); ksheehan@rcsi.ie (K.M.S.)

[9] Department of Medical Oncology, Beaumont Hospital, Dublin, Dublin 9, Ireland; osbreathnach@beaumont.ie (O.S.B.); liamgrogan@beaumont.ie (L.G.); patrickmorris@beaumont.ie (P.G.M.)

[10] Department of Pathology, The University of Texas MD Anderson Cancer Center, Houston, TX 77030, USA; WFoo@mdanderson.org

[11] Department of Surgical Oncology, The University of Texas MD Anderson Cancer Center, Houston, TX 77030, USA; YNYou@mdanderson.org

[12] Department of Radiation Oncology, St. Luke's Radiation Oncology Centre, Beaumont Hospital, Dublin 9, Ireland; Brian.O'neill@slh.ie

† Equal contribution

‡ Present address: Department of Radiation Oncology, Mayo Clinic Florida, Jacksonville, FL zip code, USA

* Correspondence: simonfurney@rcsi.ie (S.J.F.); bryanhennessy74@gmail.com (B.T.H.); krishnan.sunil@mayo.edu (S.K.)

Supplementary

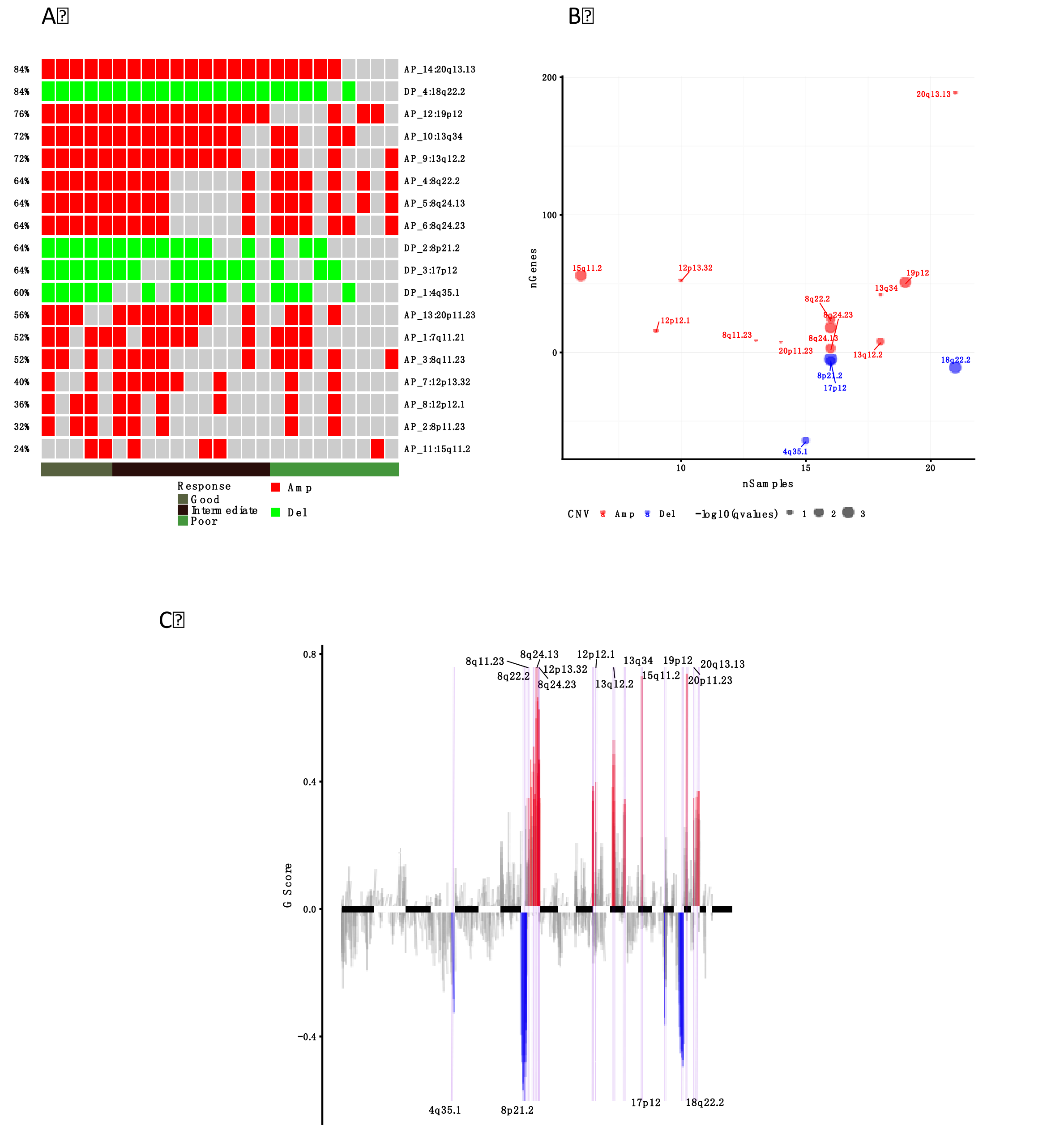

Figure S1. GISTIC2 analysis of copy number alterations. Significant amplifications and deletion peaks are shown in an Oncoplot (**A**), a Bubbleplot (**B**) and a Chromosome plot (**C**) using maftools.

Figure S2

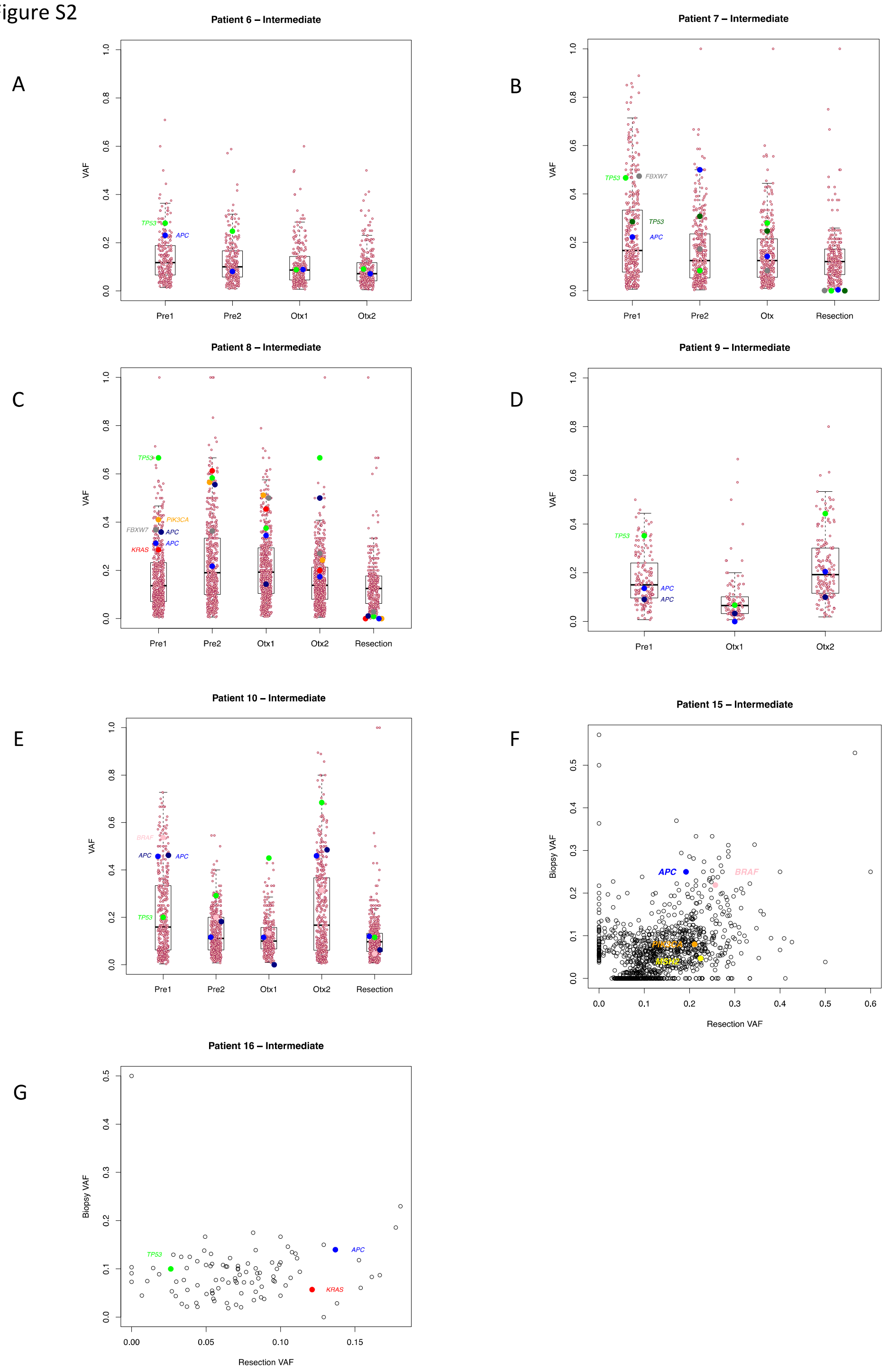

Figure S2. Longitudinal tracking of mutations in Intermediate response patients. (A–F) Patients 6–10 and 15-Boxplots of Variant Allele Frequencies (VAFs) of all SNVs (black circles) and likely driver mutations (coloured circles) in multiple tumour samples from each patient: Pre1 (Pre-treatment sample 1), Pre2 (Pre-treatment sample 2), Otx1 (On-treatment sample 1), Otx2 (Ontreatment sample 2), Resection (Surgical resection sample). (G) and (H) Patients 15 and 16—Scatterplots comparing Variant Allele Frequencies (VAFs) of all SNVs (black circles) and likely driver mutations (coloured circles) in the pre-treatment biopsy and post-treatment surgical resection tumour sample.

Figure S3

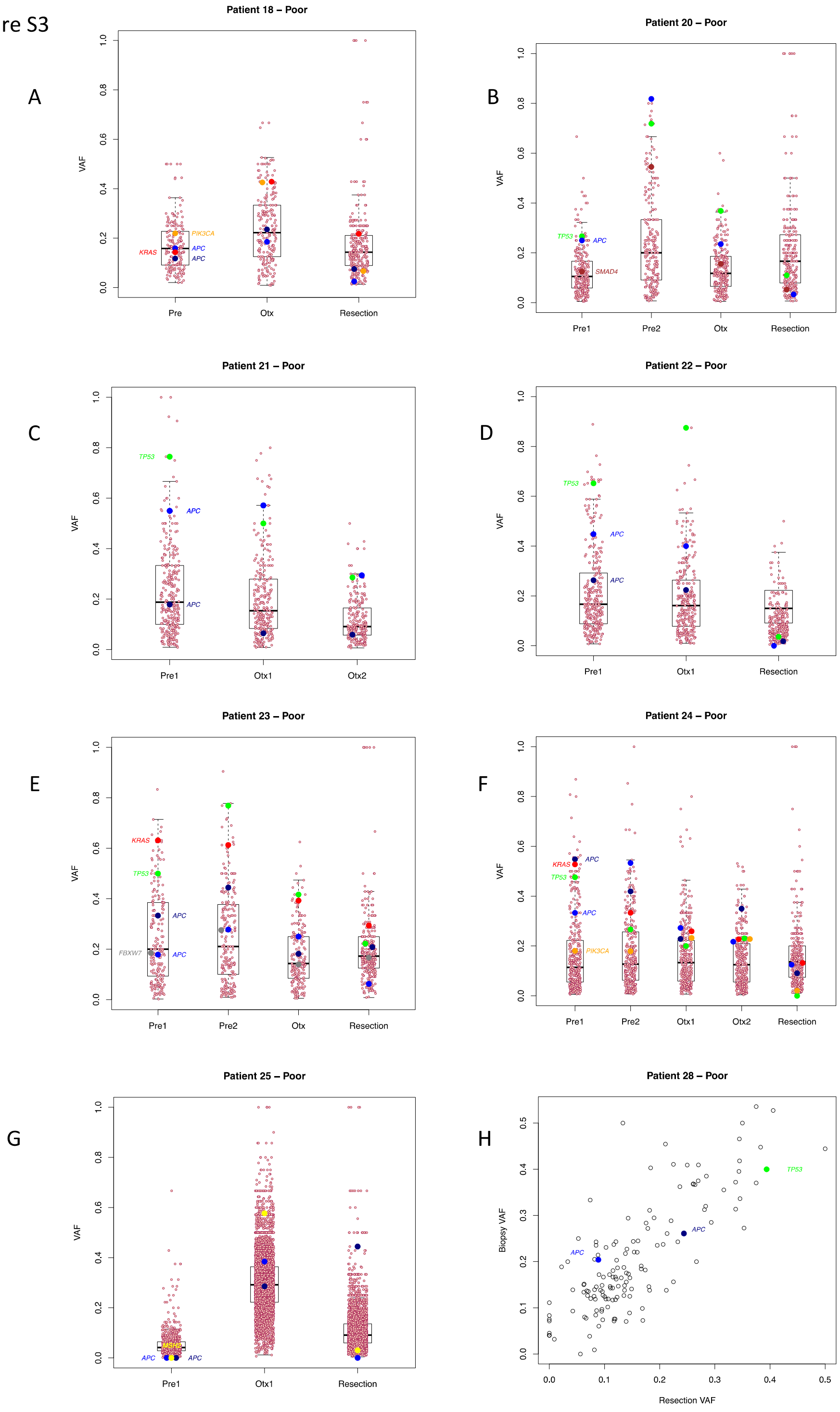

Figure S3. Longitudinal tracking of mutations in Poor response patients. (A–G) Patients 18 and 20–25-Boxplots of Variant Allele Frequencies (VAFs) of all SNVs (black circles) and likely driver mutations (coloured circles) in multiple tumour samples from each patient: Pre1 (Pre-treatment sample 1), Pre2 (Pre-treatment sample 2), Otx1 (On-treatment sample 1), Otx2 (Ontreatment sample 2), Resection (Surgical resection sample). (H) Patient 28 – Scatterplot comparing Variant Allele Frequencies (VAFs) of all SNVs (black circles) and likely driver mutations (coloured circles) in the pre-treatment biopsy and post-treatment surgical resection tumour sample.

A

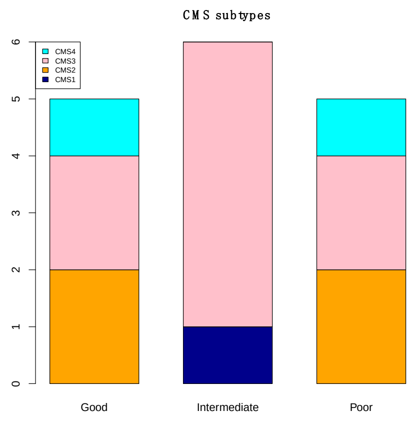

B

Patient	Biopsy	Resection	Response
14	CMS3	CMS4	Intermediate
15	CMS3	CMS1	Intermediate
16	CMS3	CMS4	Intermediate
28	CMS2	CMS2	Poor

Figure S4. Consensus Molecular Subtyping of RNA data. **(A)** CMS by response group; **(B)** CMS of matched pre- and post-NACRT samples.

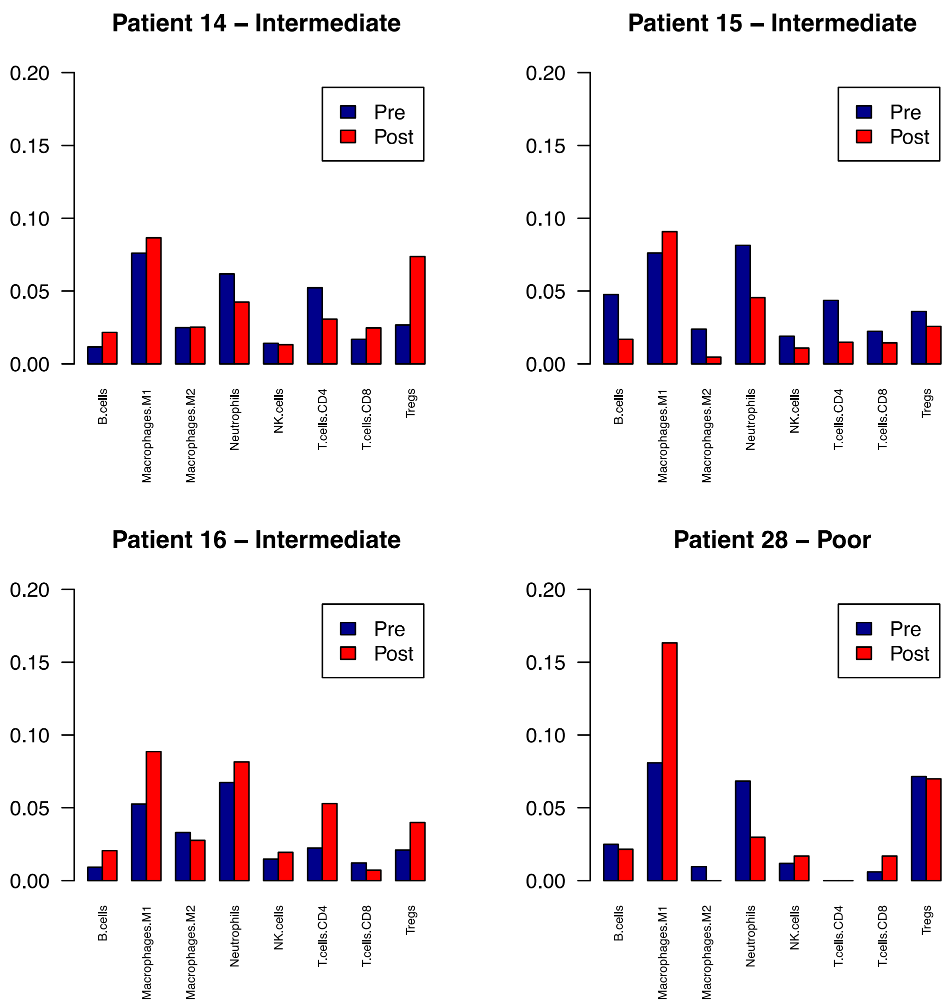

Figure S5. Immune composition of matched pre- and post-NACRT samples Immune composition of B cells, macrophages, neutrophils, NK cells, CD4+ T cells, CD8+ T cells, and regulatory T cells identified from RNA-seq data.

Table S1. Sequencing strategy summary for pre-treatment, on-treatment and post-treatment LARC samples form Beaumont Hospital (BH) and MD Anderson Cancer Centre (MDACC).

			Exome Seq			Panel Seq			RNAseq	
**Patient**	**Response**	**Source**	**Pre**	**On**	**Post**	**Pre**	**On**	**Post**	**Pre**	**Post**
1	Good	MDACC	X			X				
2	Good	MDACC	X			X				
3	Good	BH	X			X			X	
4	Good	BH	X			X			X	
5	Good	BH	X			X			X	
6	Intermediate	MDACC	XX	XX		XX	XX			
7	Intermediate	MDACC	XX	X	X	XX	X	X		
8	Intermediate	MDACC	XX	XX	X	XX	X	X		
9	Intermediate	MDACC	X	XX		X	XX			
10	Intermediate	MDACC	XX	XX	X	XX	XX			
11	Intermediate	MDACC	X			X				
12	Intermediate	BH	X		*	X		*	X	
13	Intermediate	BH	X			X			X	
14	Intermediate	BH			X			X	X	X
15	Intermediate	BH	X		X	X		X	X	X
16	Intermediate	BH	X		X	X		X	X	X
17	Intermediate	BH	X			X			X	
18	Poor	MDACC	X	X	X	X	X	X		
19	Poor	BH							X	
20	Poor	MDACC	XX	X	X	XX	X	X		
21	Poor	MDACC	X	XX		X	XX			
22	Poor	MDACC	X	X	X	X	X	X		
23	Poor	MDACC	XX	X	X	XX	X	X		
24	Poor	MDACC	XX	XX	X	XX	XX	X		
25	Poor	MDACC	X	X	X	X	X	X		
26	Good	BH							X	
27	Good	BH							X	
28	Poor	BH	X		X	X		X	X	X
29	Poor	BH	X			X			X	
30	Poor	BH							X	
31	Poor	BH	X			X			X	
32	Unknown	MDACC	X			X				
33	Unknown	MDACC	X			X				

XX indicates where more than one sample from different regions of the tumour was available for sequencing. *Indicates a further tumour was resected from this patient at a later date.

cancers

MDPI

Table S2. Significant regions of amplification and deletion from GISTIC2 analysis of pre-treatment samples at a q value threshold of 0.25.

Unique Name	Wide Peak Limits	q values
Amplification Peak 1	chr7:56173923-65425733	0.24573
Amplification Peak 2	chr8:38172779-38676913	0.21845
Amplification Peak 3	chr8:54147206-55542479	0.17629
Amplification Peak 4	chr8:96281143-100108465	0.034787
Amplification Peak 5	chr8:122627196-124749425	0.0013457
Amplification Peak 6	chr8:139767578-140714892	0.0067354
Amplification Peak 7	chr12:1-5908546	0.17088
Amplification Peak 8	chr12:24102355-26220431	0.15653
Amplification Peak 9	chr13:28519283-29004087	0.032893
Amplification Peak 10	chr13:110436580-115169878	0.17629
Amplification Peak 11	chr15:25349696-25584222	0.0013457
Amplification Peak 12	chr19:19789388-30019196	0.0013457
Amplification Peak 13	chr20:18810522-20139901	0.17629
Amplification Peak 14	chr20:42203407-58644915	0.17338
Deletion Peak 1	chr4:162431371-183694493	0.047342
Deletion Peak 2	chr8:23709669-24811556	0.00010929
Deletion Peak 3	chr17:11786796-12608339	0.015829
Deletion Peak 4	chr18:64176078-70532300	0.00053949

Table S3. Significant regions of amplification and deletion from GISTIC2 analysis of pre-treatment samples by response group (G vs IP = Good versus Intermediate plus Poor; G vs P = Good versus Poor) at a q value threshold of 0.25.

chr	start	end	length	type	pvalue	qvalue	Analysis
7	129912824	158935035	29022212	Loss	0.030769231	0.10075	G vs. IP
6	166721597	170888981	4167385	Loss	0.021978022	0.223	G vs. P

Table S4. Normalised (N_score) scores of microbial abundance from pre-treatment RNA samples and sample response group.

Name	N_score	Response	Name	N_score	Response
Actinobacteria	2.593	Good	Fusobacteria	9.432	Inter
Aquificae	0.034	Good	Proteobacteria	49.042	Inter
Bacteroidetes	21.781	Good	Verrucomicrobia	0.07	Inter
Cyanobacteria	0.131	Good	Acidobacteria	0	Inter
Deinococcus-Thermus	0.187	Good	Actinobacteria	5.762	Inter
Firmicutes	55.071	Good	Bacteroidetes	14.865	Inter
Fusobacteria	0.139	Good	Chloroflexi	0.011	Inter
Proteobacteria	19.534	Good	Cyanobacteria	0.067	Inter
Synergistetes	0	Good	Deferribacteres	0.002	Inter
Tenericutes	0.034	Good	Deinococcus-Thermus	0.657	Inter
Verrucomicrobia	0.375	Good	Fibrobacteres	0	Inter
Actinobacteria	7.167	Good	Firmicutes	38.883	Inter
Bacteroidetes	19.165	Good	Fusobacteria	1.068	Inter
Cyanobacteria	0.006	Good	Proteobacteria	38.246	Inter
Deinococcus-Thermus	0.811	Good	Spirochaetes	0.006	Inter
Firmicutes	42.926	Good	Tenericutes	0.019	Inter
Fusobacteria	0.746	Good	Verrucomicrobia	0.033	Inter
Proteobacteria	29.158	Good	Actinobacteria	7.556	Inter
Spirochaetes	0.006	Good	Bacteroidetes	19.486	Inter
Acidobacteria	0.098	Good	Cyanobacteria	0.001	Inter
Actinobacteria	11.249	Good	Deinococcus-Thermus	0.936	Inter
Bacteroidetes	15.862	Good	Fibrobacteres	0.027	Inter
Cyanobacteria	0.14	Good	Firmicutes	19.108	Inter

Deinococcus-Thermus	1.427	Good	Fusobacteria	1.029	Inter
Firmicutes	19.81	Good	Proteobacteria	51.81	Inter
Fusobacteria	1.175	Good	Actinobacteria	12.59	Inter
Proteobacteria	48.562	Good	Bacteroidetes	5.266	Inter
Verrucomicrobia	1.553	Good	Cyanobacteria	0.06	Inter
Acidobacteria	0.029	Good	Deinococcus-Thermus	2.143	Inter
Actinobacteria	10.134	Good	Firmicutes	8.695	Inter
Bacteroidetes	8.499	Good	Fusobacteria	1.181	Inter
Balneolaeota	0	Good	Planctomycetes	0.087	Inter
Candidatus_Cloacimonetes	0	Good	Proteobacteria	67.021	Inter
Candidatus_Kryptonia	0	Good	Spirochaetes	2.804	Inter
Chlamydiae	0	Good	Synergistetes	0.087	Inter
Cyanobacteria	0.045	Good	Actinobacteria	5.382	Poor
Deinococcus-Thermus	1.223	Good	Aquificae	0.004	Poor
Firmicutes	12.228	Good	Bacteroidetes	13.937	Poor
Fusobacteria	10.455	Good	Cyanobacteria	0.06	Poor
Planctomycetes	0.05	Good	Deinococcus-Thermus	0.356	Poor
Proteobacteria	57.31	Good	Dictyoglomi	0.005	Poor
Spirochaetes	0.009	Good	Firmicutes	21.844	Poor
Synergistetes	0	Good	Fusobacteria	24.727	Poor
Actinobacteria	7.596	Good	Proteobacteria	33.306	Poor
Aquificae	0.007	Good	Synergistetes	0.008	Poor
Bacteroidetes	13.644	Good	Acidobacteria	0.177	Poor
Chlorobi	0.007	Good	Actinobacteria	14.726	Poor
Chrysiogenetes	0.001	Good	Bacteroidetes	3.993	Poor
Cyanobacteria	0.018	Good	Cyanobacteria	0.181	Poor
Deinococcus-Thermus	0.208	Good	Deinococcus-Thermus	2.873	Poor
Firmicutes	69.582	Good	Firmicutes	7.006	Poor
Fusobacteria	0.171	Good	Fusobacteria	0.619	Poor
Proteobacteria	8.659	Good	Proteobacteria	70.424	Poor
Synergistetes	0.015	Good	Actinobacteria	10.457	Poor
Tenericutes	0.024	Good	Armatimonadetes	0.031	Poor
Actinobacteria	11.9	Inter	Bacteroidetes	13.538	Poor
Bacteroidetes	6.575	Inter	Chlorobi	0.055	Poor
Cyanobacteria	0.003	Inter	Chloroflexi	0.004	Poor
Deinococcus-Thermus	2.533	Inter	Cyanobacteria	0.127	Poor
Firmicutes	16.169	Inter	Deinococcus-Thermus	1.762	Poor
Fusobacteria	1.818	Inter	Firmicutes	14.655	Poor
Proteobacteria	60.962	Inter	Fusobacteria	7.804	Poor
Spirochaetes	0.039	Inter	Proteobacteria	51.55	Poor
Actinobacteria	12.087	Inter	Acidobacteria	0.001	Poor
Bacteroidetes	4.372	Inter	Actinobacteria	1.257	Poor
Cyanobacteria	0.187	Inter	Aquificae	0.002	Poor
Deinococcus-Thermus	1.528	Inter	Bacteroidetes	4.417	Poor
Firmicutes	8.666	Inter	Chrysiogenetes	0	Poor
Fusobacteria	1.302	Inter	Cyanobacteria	0.005	Poor
Proteobacteria	71.661	Inter	Deinococcus-Thermus	0.145	Poor
Synergistetes	0.113	Inter	Dictyoglomi	0.003	Poor
Verrucomicrobia	0.057	Inter	Firmicutes	9.307	Poor
Actinobacteria	3.202	Inter	Fusobacteria	73.566	Poor
Aquificae	0.001	Inter	Kiritimatiellaeota	0.001	Poor
Bacteroidetes	37.37	Inter	Proteobacteria	11.072	Poor
Chlorobi	0.001	Inter	Spirochaetes	0.001	Poor
Chloroflexi	0.015	Inter	Synergistetes	0.171	Poor
Cyanobacteria	0.01	Inter	Tenericutes	0.017	Poor
Deinococcus-Thermus	0.579	Inter	Verrucomicrobia	0.034	Poor
Firmicutes	34.344	Inter	Acidobacteria	0.028	Poor
Fusobacteria	5.415	Inter	Actinobacteria	8.276	Poor
Planctomycetes	0.03	Inter	Bacteroidetes	18.001	Poor
Proteobacteria	18.425	Inter	Cyanobacteria	0.068	Poor
Spirochaetes	0.031	Inter	Deferribacteres	0	Poor
Synergistetes	0.01	Inter	Deinococcus-Thermus	1.445	Poor
Verrucomicrobia	0.03	Inter	Firmicutes	12.041	Poor

Actinobacteria	8.813	Inter	Fusobacteria	13.583	Poor
Bacteroidetes	22.727	Inter	Nitrospirae	0.001	Poor
Cyanobacteria	0.085	Inter	Planctomycetes	0.028	Poor
Deinococcus-Thermus	1.014	Inter	Proteobacteria	46.382	Poor
Firmicutes	8.745	Inter	Spirochaetes	0.014	Poor
			Verrucomicrobia	0.028	Poor

Table S5. HR-related genes (from do Canto et al.) with somatic mutations in our pre-treatment patient cohort.

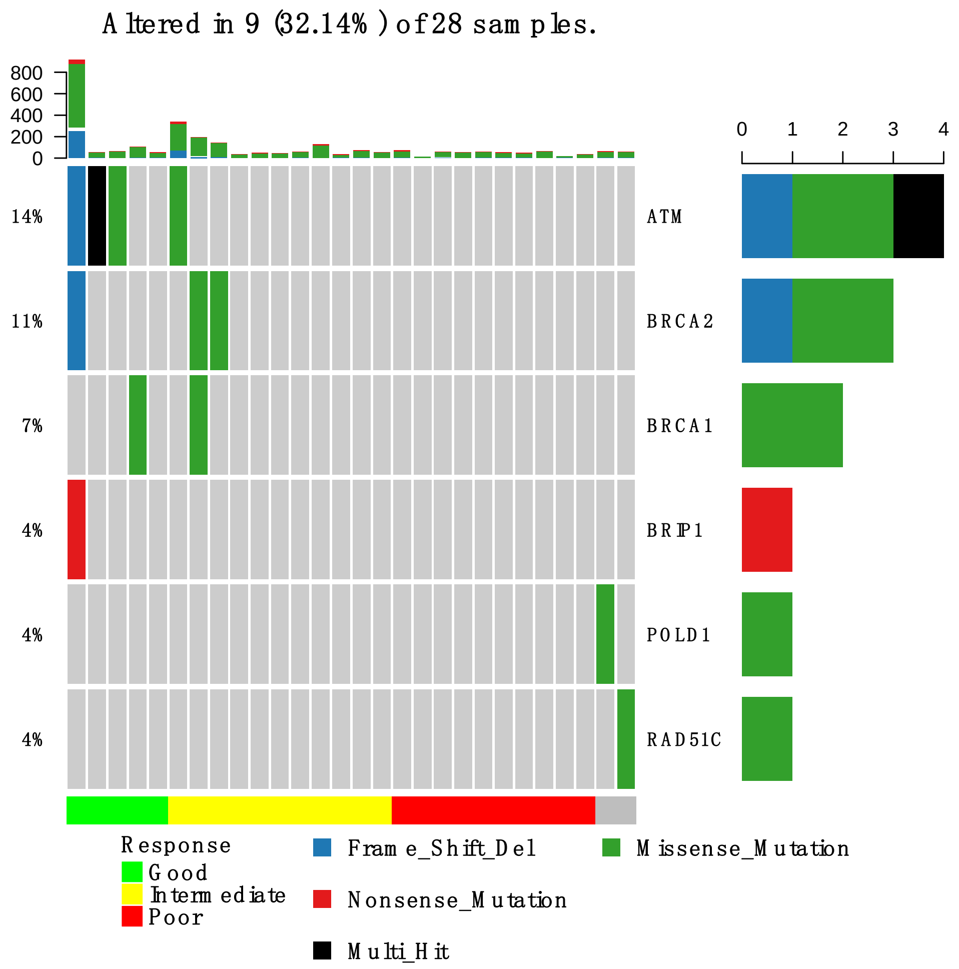

Table S6. The contribution of COSMIC mutational signature 3 (HR-associated) to each tumour genome.

Patient	Response	Sig.3
1	Good	0.050349422
2	Good	0
3	Good	0.087007485
4	Good	0.101976877
5	Good	0.322017108
6	Intermediate	0.138048115
7	Intermediate	0.27758172
8	Intermediate	0.104181945
9	Intermediate	0
10	Intermediate	0.20408252
11	Intermediate	0.380326362
12	Intermediate	0
13	Intermediate	0.091860372

15	Intermediate	0
16	Intermediate	0
17	Intermediate	0.15745017
18	Poor	0
20	Poor	0.251261784
21	Poor	0.094553676
22	Poor	0.214877578
23	Poor	0.353064566
24	Poor	0.095924513
25	Poor	0.065341746
28	Poor	0.170825376
29	Poor	0
31	Poor	0
